# Milk handling practices and consumption behavior among Borana pastoralists in southern Ethiopia

**DOI:** 10.1186/s41043-019-0163-7

**Published:** 2019-02-07

**Authors:** Kebede Amenu, Barbara Wieland, Barbara Szonyi, Delia Grace

**Affiliations:** 1 Department of Microbiology, Immunology and Veterinary Public Health, College of Veterinary Medicine and Agriculture, Addis Ababa University, P. O. Box 34, Bishoftu, Ethiopia; 2 International Livestock Research Institute, P. O. Box 5689, Addis Ababa, Ethiopia; 3 International Livestock Research Institute, P. O. Box 30709, Nairobi, Kenya

**Keywords:** Milk consumption, Milk boiling, Milk container smoking, Disease perception, Safe milk, Milk hygiene

## Abstract

**Background:**

Safety and wholesomeness of milk intended for human consumption are influenced by various interlinked factors. However, information on what these factors are, especially in the pastoral traditional communities of Ethiopia, is largely lacking. The objective of this study is to assess the hygienic milk production, processing and consumption practices, and behaviors of Borana pastoralists.

**Methods:**

The study used qualitative participatory research methods. Individual semi-structured interviews, focus group discussions, informal discussions, and observations were carried out on (1) milk handling practices, (2) perceptions of quality and safety of milk, including perceived criteria for good milk, (3) awareness of milk-borne diseases, and (4) perception towards milk boiling practices. The interviews and discussions were audio recorded, transcribed, and analyzed by identifying themes.

**Results:**

Some risky behaviors related to milk handling and consumption were identified. These include unhygienic conditions in handling milk and milk products, consumption behaviors such as consuming raw milk purchased from markets, and children directly consuming milk from the udder of animals (e.g., goats). There was a very strong reluctance to boil milk before consumption mainly because of the misconception that nutrients in the milk are destroyed when milk is boiled and “boiled milk is dead”. On the other hand, potential risk mitigation practices were identified such as smoking of milk containers (which may help reduce microbial growth), processing milk through fermentation, consuming milk in boiled tea, and a recent trend towards boiling milk for babies. However, the latter was not motivated by concern over microbial hazards but the belief that raw milk could form curds in children’s stomach which might then suffocate them.

**Conclusion:**

The findings highlight the need to promote hygienic handling practices of milk and closely engage with local communities to improve their understanding of milk safety to facilitate change in practices. Educating pastoralists on good milk production practices should be given priority. One of the ways to do this could be by strengthening the integration of milk hygiene in research and development programs as an entry point for behavioral change towards the safe handling and consumption of milk and milk products.

## Background

Microbial contamination of food caused by improper handling and poor environmental hygiene and sanitation is the leading cause of foodborne morbidity and mortality, especially in developing countries [1, 2]. In addition to causing morbidity and mortality, foodborne diseases affect health and nutrition outcomes of human beings in several ways. For instance, foodborne diseases can result in poor appetite thereby reducing dietary intakes required by individuals. They can also cause malabsorption and reduced utilization of micronutrients due to diarrhea and vomiting [3]. Food can be a vehicle for a number of pathogens belonging to bacterial, viral, and parasitic agents, including bacteria responsible for the majority of foodborne illnesses [4, 5]. The most common bacterial foodborne pathogens include *Salmonella* spp., *Escherichia coli* O157:H7, and *Campylobacter* [5]. *Salmonella* infection is a major health problem both in developed and developing countries. Specifically, non-typhoid *Salmonella* spp. are responsible for a number of health problems in humans such as gastroenteritis, bacteremia, and subsequent focal infection [6, 7]. These types of infections could be highly problematic especially in immunocompromised individuals [7]. Majowicz et al. [8] estimated that globally, *Salmonella* infection is responsible for 93.8 million cases of gastroenteritis and 155,000 subsequent deaths per year. There are a variety of animal source foods associated with *Salmonella* infection in humans. Some of these foods are ground beef, chicken, eggs, and unpasteurized dairy products [9]. *Escherichia coli* O157:H7 is another cause of foodborne diseases that cause life-threatening sequelae such as hemolytic uremic syndrome (HUS) and thrombocytopenic purpura [10]. Transmission to people occurs primarily through ingestion of inadequately processed or contaminated food or water [11]. *Campylobacter* is another common bacterial foodborne pathogen that affects humans and resulting in a range of symptoms from mild to severe bloody diarrhea [12].

Milk is a valuable source of both macro and micronutrients. On the other hand, milk is highly perishable and can lose its quality and safety within a short period of time if not handled under hygienic conditions [13]. As a result, milk can be the source of dangerous pathogens to consumers leading to serious health problems [14–16]. As such, microbial contamination of milk and dairy products constitutes an important health risk for consumers [17]. Milk directly obtained from a healthy udder is considered to be sterile, and most microbial contamination of milk and milk products occurs during milking, storage, transportation, and processing [18]. The safety and wholesomeness of milk intended for human consumption are affected by a number of complex and interlinked factors [19]. Contaminated milk can harbor a variety of pathogenic microorganisms such as *Salmonella* spp., *Escherichia coli* O157:H7, toxigenic *Staphylococcus aureus*, and *Listeria monocytogenes*; all of these cause significant human illnesses [14, 20–23]. In addition, raw milk can cause infection by classical zoonotic agents such as *Mycobacterium bovis*, *Brucella* spp., and *Coxiella burnetii* [24].

Even though detailed information on the impact of zoonoses is not available, milk-borne pathogens are of public health concern in many developing countries. For instance, a study conducted in Mali found an increased risk of food-related intoxication characterized by diarrhea or vomiting in children consuming milk products [25]. Similarly, Darapheak et al. [26] showed an increased risk of diarrhea in children consuming milk in Cambodia. An observational epidemiological study by Kaindi et al. [27] in Kenya reported that camel milk and vegetable market chains pose the greatest risk for foodborne gastrointestinal illnesses (diarrhea and/or vomiting). In pastoral communities, milk is widely consumed in raw form and makes a substantial contribution to protein and micronutrient requirements of the community [28, 29]. The trade-offs, however, are health risks that come with poor hygienic practice of milk handling and consumption.

Ways to ensure the quality and safety of milk include good hygiene of the milking environment, using food-grade containers (for example, stainless steel which is easy to clean), cooling the milk immediately after milking, and boiling or pasteurization before consumption [30]. Such practices are not common in traditional smallholder or extensive livestock production in developing countries like Ethiopia [31]. As a result, milk is produced under unhygienic conditions leading to high microbial contamination and spoilage with associated health risks to consumers [32–34]. In Ethiopia, milk is produced under urban/peri-urban, crop-livestock, and pastoral/agro-pastoral livestock husbandry systems. In the country, through traditional fermentation system, milk is converted into different products such as whole fermented milk (*ergo*), curd milk with whey partially removed (*ititu*), soft cheese (*ayib*), and butter [35]. The quality and safety of the products are highly variable with high health risks [36].

To work towards raising awareness and designing acceptable interventions to bring about change in the behavior of people involved in milk production and handling, it is important to understand the local context of milk production, handling, and processing. In this respect, participatory action research involving qualitative investigation is well placed to identify and implement appropriate risk reduction strategies and consequently reduce the health risks associated with particular foods [37]. So far, little work has been carried out on milk hygiene in pastoral areas in Ethiopia. Especially, studies on the perception and practice of people towards milk handling and processing are lacking for pastoral livestock production systems. Therefore, this study set out to assess the behavior of people with regards to milk production, processing, and consumption using qualitative participatory research methods with the goal of using the findings to develop appropriate educational programs for pastoralists on improving milk handling practices.

## Materials and methods

### Study area

The study was carried out in Yabello district of Borana zone located in the Oromia regional state of southern Ethiopia at approximately 570 km from Addis Ababa. Four village administrations—Dharito, Elweya, Surupha, and Did Yabello—were selected and included in this study based on milk production potential of the villages and alignment with other ongoing animal health research projects implemented by the International Livestock Research Institute. Surupha is largely inhabited by the Gabra ethnic group and the other three villages are inhabited by Borana. Both ethnic groups share similar cultures and lifestyle and speak the same language—Borana dialect of Afan Oromo, which is a family of Afro-Asiatic languages. The only exception is that Gabra are mostly Muslims and Borana follow traditional religions. In Borana, several households belonging to the same sub-lineage or tribe reside in clusters or neighborhoods called *olla*. The livelihood of the people is largely dependent on livestock production. Historically, Borana people were cattle keepers but have started diversifying by keeping different livestock species. For example, keeping camels was not common in Borana but is becoming more common nowadays. Traditionally, camels are regarded as belonging to the Somali ethnic groups living in areas bordering Borana [38].

### Study framework and data collection methods

The framework applied to investigate practices and perceptions is based on the general principles of good dairy production practices [39]. It takes into account that the quality and safety of milk could be affected by a number of factors along the production and processing chains [19]. Based on this, it covers key principles of good dairy production practices which ensure that milk and milk products are safely produced. It also considers the nutrition and health status of the dairy animals, proper milk collection, storage, processing, and consumption [17]. The social and cultural context of food producers, handlers, and consumers also plays an important role in ensuring the safety of the products [40, 41]. To cover these aspects, a qualitative research approach that allows data collection on how people perceive practices was needed.

As described elsewhere [41], different qualitative participatory data collection methodologies which included individual semi-structured in-depth interviews (IDI), focus group discussions (FGD), and direct observations were used. The question guide addressing the framework outlined above was initially drafted in English and translated to Afan Oromo (Borana dialect). A total of 40 women (10 in each village) were individually interviewed using a pre-tested semi-structured question guide. The main source of livelihoods for the women who participated in the IDIs is traditional livestock keeping. Four FGDs, one in each village, were also carried out with six to eight women. All the women, both in the IDIs and FDGs, had no formal education. The IDIs and FGDs were carried out with the help of a female field assistant working in the area as animal production expert. The IDIs and FGDs were audio recorded and field notes were taken to supplement the recordings.

Overall, the qualitative data collection focused on (1) milk production and processing, (2) perceived criteria for good or bad milk in terms of safety, and (3) perception on milk boiling and consumption practices. Categories defined for data collection were based on technical and social/cultural aspects of food safety research. Information collected on milk production and processing was put under technical categories, while information on the perception of the people in selecting the forms of milk they consume and their risk mitigation mechanisms was captured under socio-cultural categories. This approach was derived from Fischer et al. [40], who suggest the combined use of natural and social sciences to sufficiently improve the safety of food in domestic environments.

Similar topics were addressed through the different data collection tools used in this study (IDIs and FGDs), which allowed some triangulation. In FGDs, more emphasis was given on milk boiling and consumption practices instead of processing. Due to difficulty in directly translating the scientifically understood terms of “microbiological quality or safety,” we used general questions such as “what makes milk bad?” or “what qualifies milk as good?” for the assessment of the quality and safety of milk.

Before the qualitative data collection, verbal consent was obtained from each of the respondents by explaining the objectives of the study. The information collected was what is normally freely shared among pastoral communities and written consent was not sought.

### Data analysis

The audio recordings of the qualitative data were transcribed verbatim, with the exception of repetitive terms or ideas. The transcription was done by the first author who listened to the recordings and translated them into English. If no exact word or description was available in English, the term in Afan Oromo was used by putting it in brackets to minimize loss of ideas or concepts during translation.

Data analysis process suggested by Green et al. [43] was used in this study. This process includes immersion in data, processing of codes, creation of categories, and identification of themes. Accordingly, the transcripts were repeatedly examined, ideas were grouped into the themes used in the questions guide, and new themes were added as appropriate. This interactive process also included findings from field notes. The first author of this paper was leading the FGDs and facilitated the identification of emerging themes during the IDIs. For example, information regarding the relationship between smoking of milk containers and the quality of milk was one of the themes that emerged during data collection. The themes were coded using free software QDA Miner Lite v1.4.3, Provalis Research [42]. To portray the qualitative data, different quotes in the words of the respondents were highlighted. Pictures were also taken to illustrate the different milk handling and consumption practices.

## Results

### Preference of pastoralists for milk of different livestock species

People in the study area keep different species of livestock for milk production purposes. Results of the study show differences in preference for milk obtained from different livestock species. Cow milk was widely produced and had high cultural values due to the ease of converting it into different dairy products such as yoghurt and butter. One of the reasons why the pastoralists preferred cow milk was for the cosmetic use of butter derived from cow milk as hair treatment. The following statements made by the pastoralists illustrate this view.

I churn cow milk to get butter that can be used as hair treatment. (IDI 1, 33 years old, pastoralist)

Milk from camels can’t be processed into butter. It’s only used for drinking. Cow milk, on the other hand, can be converted into butter and used for hair dressing. It also has cosmetic value. (IDI 25, 35 years old, pastoralist)

The level of production of camel milk varied across the surveyed villages. In Surupha village, which is mostly inhabited by Gabra ethnic group, camel milk was produced in large volumes and much of the fluid milk was marketed by transporting it for long distances to the Kenyan border. On the other hand, consumption of camel milk was considered a taboo by some Borana clans like Qallu Karayu. The following quotes are taken from the pastoralists expressing the cultural taboo associated with camel milk consumption.

People have not consumed [camel milk] for a long time. They consider camels as dirty and because of that they do not consume [camel milk]. People who own camels do not drink the milk even with tea. People in our village (Olla) do not consume camel milk. Our village is inhabited by Karayu clan. (IDI 8, 40 years old, pastoralist)

We’ve inherited this from our ancestors. We are Qallu and Qallu people don’t consume milk from camels. (IDI 7, 50 years old, pastoralist)

In all the villages included in this study, goat milk was also produced but in small volumes. It was appreciated for its nutritional value and was often consumed by children directly from the udder or by mixing it with boiling tea. On the other hand, there was a complaint of bad odor of goat milk by some respondents.

Milk from goats has odour similar to the goats themselves. Milk from goats is also small (low quantities). (IDI 2, 43 years old, pastoralist)

In Borana, sheep were not milked and consequently, the pastoralists are not accustomed to consuming sheep milk.

### Milk processing into different products

**Fig. 1:**
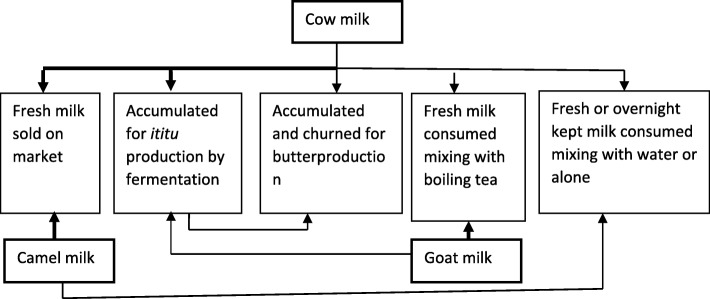
Milk production and processing (the thickness of lines reflects the volume of milk)

Cow milk was processed into different products such as yoghurt, butter, ghee (melted and filtered butter), and butter milk. Goat milk was not commonly processed into such traditional dairy products. Camel milk was also not commonly processed into other dairy products; instead, the fresh milk was consumed directly without any treatment or was sold on the market. Figure 1 below summarizes the different forms of milk processing and consumption of the pastoralists.

*Ititu* (traditional yoghurt) is fermented whole milk prepared by accumulating milk for several days or weeks and continuously removing the whey (the fluid part). According to the respondents, yoghurt is a stable dairy product which can be prepared over 7–30 days by continuously adding fresh milk and simultaneously removing the whey. Preparation of yoghurt starts with cleaning and smoking of the container. Thereafter, fresh milk is added to the container and allowed to curd through natural fermentation without using any starter culture. Depending on the environmental temperature (i.e., season of the year), the first batch of milk can curd between 1 and 3 days. After formation of the curd, the whey is removed by inserting a tube-like wood called *dhuyyuma* into the curd milk, and the whey is sucked out by mouth or the milk storage container is turned upside down to allow the fluid part to flow out. Every time fresh milk is added and the whey removed, the lid of the container is cleaned and sometimes fumigated with smoke.

**Fig. 2:**
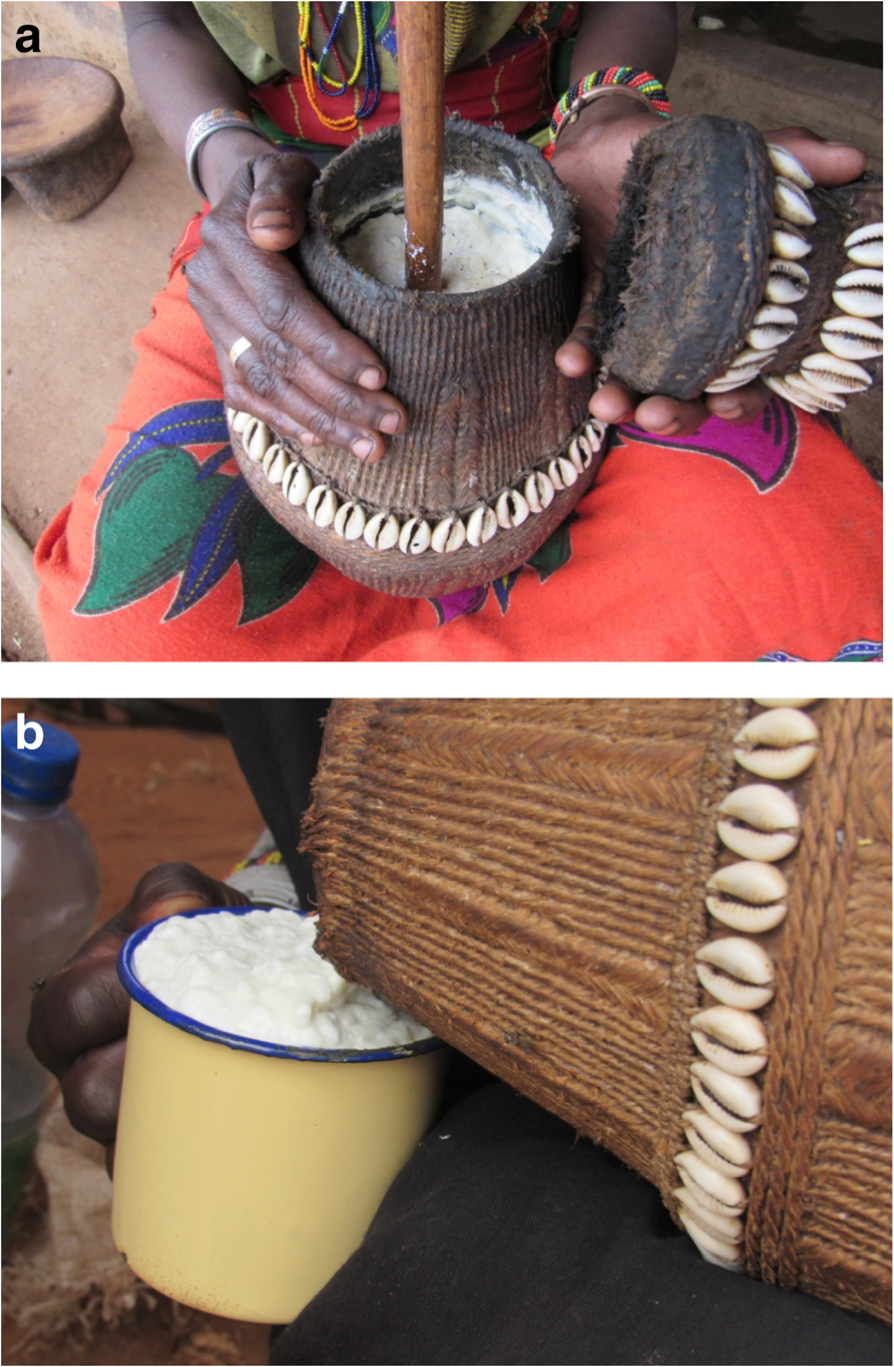
**a** Whey removal from serially accumulated curd milk during preparation of yoghurt by inserting *dhuyyumaa* and sucking out whey. **b** Dispensing of yoghurt on market for selling

Smoking of utensils used for milking or storage was reported as a very common practice. The containers are smoked by either turning them upside down on burning wood or inserting chips of burning wood into the container and continuous spinning of the container until the smoke dies. Alternatively, the burning end of a wood can be continuously rubbed against the internal wall of containers to achieve the same effect. Depending on the duration of preparation, the consistency of the yoghurt can vary from semi-fluid to semi-solid (Fig. 2). The traditional yoghurt sometimes has an extremely sour taste, and in this case, the pastoralists mix it with table sugar or butter ghee to increase its palatability. The product is served in a small cup which is shared among family members. Yoghurt is widely liked by the pastoral people and often served to the household head or to special guests. It is also served during festivities such as *Jila*.

Similar to yoghurt, processing of milk into butter starts with cleaning and fumigation of containers used for churning. The milk is accumulated and allowed to curd for 2–4 days depending on the volume of milk produced and the season. After that, the curd milk is churned by moving the container back and forth for several hours. Finally, the butter is removed by hand and put in a plastic or other container and kept for sale, home consumption, or used as hair treatment by women. The butter is converted into ghee by melting and separating the fat from the non-fat solid. Pastoral women stated that ghee production nowadays is not a common practice in the area given that marketing of fresh milk has become more popular. Butter milk is produced and used for human or animal consumption depending on the season and availability of milk (often produced during wet season).

### Milk handling and consumption behavior with risks to human health

#### Hygiene in milk handling practices

The pastoralists know that post-milking handling and processing practices can affect the hygienic quality of milk and milk products. They believe that milk from “healthy animal” is “healthy” and most contamination and subsequent lowering of the quality of milk happens after milking.

We [Borana community] believe that milk in the udder has no harm. (IDI 14, 35 years old, pastoralist)

If humans don’t make it bad, milk cannot be bad. (IDI 2, 43 years old, pastoralist)

In some cases, it was also noted that udder health is a contributing factor for poor quality of milk.

The milk has ‘disease’, when the udder is ‘diseased.’ (IDI 13, 50 years old, pastoralist)

Women were responsible for handling and processing of milk or milk products, which was stated by FGD participants as indicated below.

The quality of milk is within the hands of women [women are responsible for hygienic keeping]. (In Afan Oromo: Midhaginni aannanii, harka nadheeni keessa jira) (FGD 43)

Irrespective of the acknowledgements of the importance of hygiene in milk production and processing, observation of milk handling and processing practices revealed apparent unhygienic conditions. For example, there was no attempt by the pastoralists to remove dirty matter from the udder before milking. Hand milking was used, and the persons milking the animals were observed not to wash their hands before milking or between milking of different animals in a herd. Lactating animals were housed in kraals full of manure.

Borana pastoralists often use traditional containers for milking, storage, or transportation of milk. They have also started using other containers such as plastic jerrycans for milk transport or storage. Both traditional containers and plastic jerrycans are difficult to clean.

#### Milk boiling and consumption behavior

**Fig. 3:**
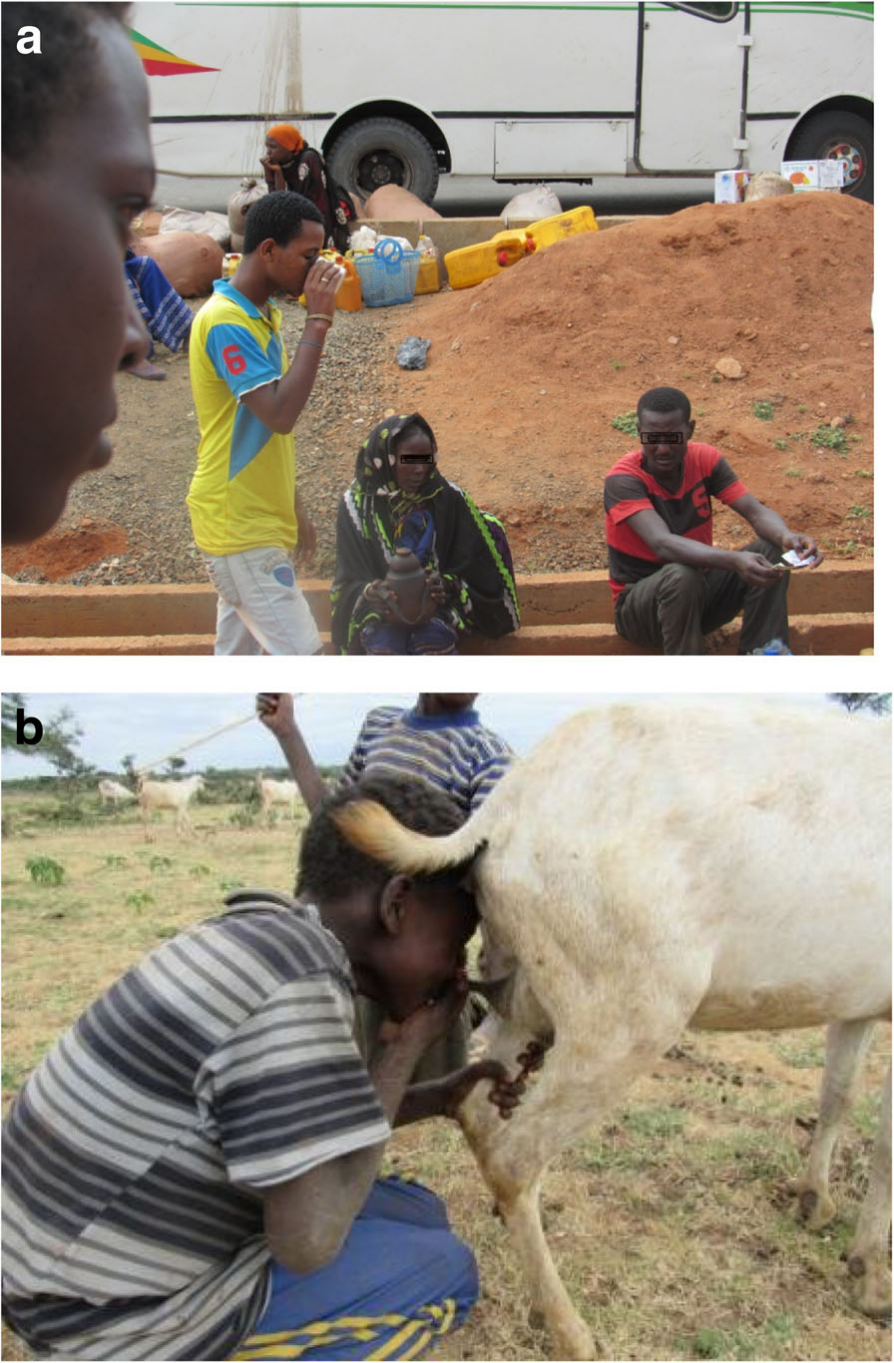
Risky milk consumption behavior. **a** Raw milk purchased from market and directly consumed without any treatment. **b** Milk consumption directly from udder of goat by children in Borana (a common practice during herding)

Milk is consumed by Borana pastoralists in different forms which include fresh raw milk soon after milking, raw milk kept overnight at room temperature, or milk processed into different products. As indicated above, the main means of processing milk into different products is by natural fermentation. But this study also revealed that raw milk consumption is very common in Borana. For example, it was observed that people buy raw milk and consume it on the spot during market days (Fig. 3a) and sometimes children consume goat milk directly from the udder (Fig. 3b).

Other practices observed that could potentially impair the health of consumers include selling raw milk for direct consumption on market days. The same cup, after being rinsed with unclean water, was shared among different customers for drinking milk or yoghurt. Potentially high microbial contamination was observed especially during the selling of yoghurt. For example, due to the semi-solid nature of the yoghurt, pouring is not easy when selling in markets which means the women selling used their hands to assist pouring. Moreover, the milk selling points were mainly on dusty road sides or the side of big livestock markets, making microbial contamination easier.

Boiling of fresh milk was not a common practice in the area. The main reasons given for not boiling milk were the long-time tradition of Borana people for not doing so and the perception that boiling milk destroys nutrients—boiled milk is dead milk.

Fresh milk has more benefits; the raw milk, you say ‘raw’. Boiled milk has no taste. We don’t like it. When boiled for children, not tasty. Even children don’t like boiled milk; they’re accustomed to raw milk. Boiled milk has no ‘qarruu’ (thick, creamy part of milk on top). That is why we don’t like boiled milk. Has no ‘qarruu’ and does not give any [nutritional] benefit. If you smoke containers and drink raw milk and give raw milk to children, there is a quick nutritional improvement in children. The same is true for adults. Boiled milk is not nutritious. That is why we don’t want to boil milk. (FGD 3)

We don’t boil [milk]. In other places, [milk is] boiled for children. When boiled, vitamins are destroyed. Milk has ‘qarruu’ (cream). When ‘ititu’ is prepared, when churned what becomes butter is the cream. When boiled, the vitamin is destroyed as we say in Borana. Smoking alone makes milk good. Milk in which cream has been removed is not good. (IDI 36, 37 years old, pastoralist)

We want raw milk. Boiled milk is dead. Raw milk is good. Only educated people boil milk. (IDI 28, 33 years old, pastoralist)

#### Poor awareness regarding milk-borne diseases

The pastoralists showed low awareness of milk-borne diseases. On the other hand, the participants often emphasized the nutritional and medicinal value of consuming milk.

One who doesn’t drink milk will get disease. The bone of the one who drinks milk gets strong and [the person physically] becomes attractive. The one who doesn’t drink milk becomes dry [referring to skinny] like me [referring to herself]. (IDI 7, 50 years old, pastoralist)

The milk itself is medicine. Fresh milk can be recommended for TB [tuberculosis] patients. (IDI 8, 40 years old, pastoralist)

We haven’t seen sickness on this. We haven’t seen any problem with the milk from our animals. We use it to raise our children. We don’t know one can get disease from milk. (IDI 11, 35 years old, pastoralist)

We don’t think so![referring to her opinion that milk cannot be a vehicle for pathogens]. We have been drinking milk our whole life (IDI 18, 20 years old, pastoralist)

On the contrary, in some interviews and discussions, pastoralists mentioned human health problems as a result of the consumption of milk or milk products. Gastritis as a result of consumption of soured milk, general gastrointestinal disturbances, delay in wound healing when drinking milk stored in a non-smoked container, and brucellosis (“*sallessa*”) were among the health problems mentioned. The following quotes refer to different health problems perceived to be associated with the consumption of milk or milk products.

Gastritis:

People drinking soured milk can get stomach problems. (IDI 28, 50 years old, pastoralist)

General gastrointestinal problems:

There is a child who was sick and when taken to the clinic, health professional said it is from cow milk (IDI 30, 17 years old, pastoralist)

One can get disease if not accustomed to drinking milk. For example, if you are given milk, it passes through you [diarrhoea] (IDI 35, 27 years old, pastoralist)

Delay in wound healing:

In Borana there was a saying in the past that when a wound is not healing quickly, the person had consumed milk from a non-smoked container. (IDI 36, 37 years old, pastoralist)

Milk from non-smoked container is a problem for children. It causes slow healing of leg wounds. The wound is difficult to heal. (FGD 1)

Brucellosis:

There is one disease which is acquired from milk. From milk of aborted animal [‘salleessa’]. One can get [the disease] from ‘salleessa’ milk. One can be without hand [probably referring to paralytic situation]. I had this kind of problem in my family. The patient was treated several times and cured. It [the disease] is called ‘salleessa’ milk. Whey milk from ‘salleessa’ given to children causes disease. The case was a long time ago. The girl is now in 4^th^ grade. For about 6 years, the girl was unable to go to the toilet. She was taken to Moyale Hospital and they said it is disease of milk and she recovered after 22 injections. (FGD 2)

Different categories of perception regarding milk-related health problems among participants in the IDIs are summarized and depicted below (Table 1). Of 40 informants participating in the IDIs, 25 believed that milk cannot transmit any disease.

**Table 1:** Summary of awareness of pastoralists regarding risks of milk-associated health problem as a result of consuming milk (from in-depth interviews)

Category of awareness	Number of informants
Consumption of milk in any form has no negative health impact	25
Consumption of soured milk or milk products exacerbates gastritis	3
Consumption of milk stored in non-smoked containers delays healing of wounds	2
Consumption of raw milk causes formation of curd in children’s stomachs and upon vomiting, may suffocate the child	1
Consumption of milk causes general gastrointestinal disturbance	6
Undetermined response	3

### Potential local health risk mitigation strategies associated with milk handling and consumption

#### Fermentation and smoking of milking and storage containers

The pastoralists have a strong belief that proper smoking of milk utensils is an important way of ensuring the good quality and safety of milk and dairy products. Many of the study participants mentioned smoking as the best way to ensure quality and shelf life of milk and traditionally produced dairy products. According to the respondents, lack of proper smoking of containers leads to milk spoilage. Besides increased shelf life of milk, the pleasing flavor of the products was mentioned as a reason for smoking containers.

The following quotes from the interviews and discussions describe the importance of container smoking in ensuring the quality of milk.

If you don’t smoke milking vessels or storage containers, milk curds quickly and becomes sour. If you smoke storage containers but not milking vessels, milk goes bad. If you smoke both, both will have good aroma and you add good aroma to the milk. (IDI 25, 35 years old, pastoralist)

What makes [milk] bad is containers which have not been properly smoked. If the container is sufficiently smoked, milk is not spoiled. If the storage containers and milking vessels have not been properly smoked, putting milk into foul-odor containers can result in disease. Smoking [container] has benefit; it gives good flavor to the milk. Containers which haven’t been sufficiently smoked make milk bad. (FGD 1)

#### Boiling milk for specific age groups and mixing milk with boiling tea

Borana pastoralists boil fresh milk for babies younger than 1 year. The main reason for this was to prevent milk from curding (*qullichoo*) after ingestion. According to the pastoralists, when children are given raw milk, the milk curds in their stomach and this can cause suffocation if the child vomits. Therefore, the milk should be boiled to prevent possible choking upon vomiting.

The following quotes explain in detail the perceptions of the pastoralists regarding milk boiling for children.

For small children milk is boiled. Adults drink as it is. When we visited the health centre, we were told to boil milk we give to children. So, milk should be boiled and cooled, the cream lining on the surface (‘qarruu’) removed. When children are given raw milk and when they vomit, ‘qullichoo’ (curdled milk inside stomach) is formed and this can block the baby’s esophagus. If the milk is boiled and given to children, ‘qullichoo’ is not formed. (FGD 1)

The reason [milk is] boiled for children is ‘qullichoo’, formed when vomiting. If boiled, [there is] no [formation of] ‘qullichoo’. ‘Qarruu’ (creamy part) is removed and the milk is given to children. ‘Qulichoo’ can suffocate children when vomiting. Adults can remove [the milk curd when vomiting]. In adults, no problem! The stomach is accustomed with raw [milk]. (FGD 4)

Milk is boiled and given to children. [The boiling] prevents ‘qullichoo’. When children drink raw milk and vomit, [it is] difficult [for them] to expel the milk curd. (IDI 39, 23 years old, pastoralist)

It was further mentioned that after boiling, milk was diluted with water as described below.

For small children, I have one baby, I boil the milk in a pot and dilute the boiled milk by mixing it with pure water. (IDI 11, 35 years old, pastoralist)

It was mentioned that milk (especially goat milk) is also consumed by adding it to boiling tea. The following response of an interviewee described this.

You drink the milk with tea; whole milk is not consumed. The tea itself is boiled and the milk is added when the tea is still hot. Then you drink it. (IDI 11, 35 years old, pastoralist)

### Changing trends on milk handling and health risk perceptions

The study found that there have been recent changes in the perception of the pastoralists regarding milk quality and safety. Health extension and research activities in the area may have resulted in these recent changes in perception. The quotes below support this assumption.

Once upon a time they [referring to animal health researchers] came to our kraals and showed us, by milking something red [making a sign of shaking a container] and it precipitated. The ones with blocked teat, they opened. The ones with udder problem, the milk curdled (clotted). Though we observed this before, we continued drinking [without boiling] except for children. After we saw this, we stopped [giving raw milk] for children. On this particular, day we observed and understood that cow milk is a disease [to say cow milk carries disease]. Those people drinking milk have disease. The people were veterinarians who were monitoring herd. I had about 12 lactating cows and out of them, only three cows were found to be healthy when examined. As a result, I have concluded that cow milk is a disease. (FGD 2)

We have been told that cattle acquire disease and the milk is not suitable for children. The health professionals are telling us that. They said milk should be boiled before it’s given to children. As a result, we are boiling milk for children. (FGD 2)

Boiling milk for children is a very recent practice after we have been told by health professionals to do so. (FGD 1)

## Discussion

This study used qualitative research methods to assess the behavior of pastoralists towards safe and hygienic production, processing, and consumption of milk. It also tried to capture the reasons behind these behaviors. Current practices observed in Borana fit with common behaviors that expose milk to bacterial contamination. These include compromised health of the dairy animal, unhygienic milking environments, unclean milk containers, contaminated water used for washing containers and other milk utensils, and inadequate precautions taken by humans handling the milk [43]. Fischer et al. [40] stated that unsafe food consumption is the result of the combined effects of the actual practices in food production, processing and consumption, and perceptions of people (the psychological aspect). Hence, controlling bacterial contamination necessitates a comprehensive and systemic approach to address the multiple layers of physical and psychological causes. Regarding this, Fischer et al. [40] further stated that food safety improvement programs designed to address the technical aspect only may fail and stressed the approach of integrating messages that address the social and technical aspects of food safety in public health education programs.

The processing of milk into different traditional products through natural fermentation described in this study is similar to findings of previous studies in Ethiopia and elsewhere [33, 35]. Volume of cow milk consumed in different forms can vary depending on the season of the year. During the rainy season (February to March), surplus milk can is usually available and the pastoralists process it into different products, such as fermented curd milk, butter, and butter milk.

Direct consumption of milk from the udder of goats practiced by children can put them at risk of getting dangerous pathogens such as *Brucella melitensis*. Preference for raw milk is not restricted to the traditional communities of developing countries like Borana pastoralists. In some cases, people in developed countries also advocate raw milk consumption claiming better nutritional qualities, taste, and health benefits [14]. But benefits of raw milk consumption cannot be easily substantiated; the claimed benefits are simply myths [22]. Studies showed that raw milk consumption is risky for human health even when produced under hygienic environments [44]. Raw milk has been implicated in a number of foodborne disease outbreaks worldwide [24].

Raw milk consumption and unsafe handling of milk can put consumers at risk for milk-borne zoonotic infections [45, 46]. The habit of raw milk consumption in the study area can be a health hazard for the pastoral community, given that the area is endemic for zoonotic diseases such as brucellosis and tuberculosis. For example, Duguma et al. [45] reported 3.8% prevalence of bovine tuberculosis in the area, and this can be a high health risk coupled with poor awareness of the pastoralists about the transmission of the disease to humans. Similarly, Megersa et al. [46] reported seroprevalence of brucellosis in 10.6% of cattle, 2.2% of camels, and 1.9% of goats in Borana.

Pastoral women largely were unaware of milk-borne diseases. Instead, they emphasized the advantages of consuming raw milk. Milk-related health problems were mentioned only a few times, and these were mostly not directly related to microbiological safety. This can be either due to the adaptation of the local communities to unhygienic raw milk consumption or presence of effective risk mitigation strategies. It is known that a repeated low-dose exposure to pathogens in food or water of poor microbiological quality can reduce the associated diseases in communities compared to those irregularly exposed to the pathogens [47]. While the pastoralists may have adapted to milk or milk products of poor hygienic quality to some extent, improving hygiene is still required due to some emerging challenges and opportunities. This is especially true due to the fact that selling of milk and milk products is becoming a significant income source for pastoral women and non-pastoral people are becoming important milk buyers. This situation necessitates provision of milk of good microbiological quality to the markets.

Some practices revealed in this study, such as smoking of milk containers and processing of milk through fermentation, can be considered as risk mitigation strategies with the potential to reduce the incidence of milk-associated illnesses. In a laboratory-based experiment, smoking of milk containers in the production of homemade yoghurt improved microbiological quality and taste compared with the use of non-smoked containers [48]. A recent study in Kenya also demonstrated the effectiveness of smoking containers in preventing microbial growth and subsequently improving the keeping quality of camel milk [49]. It is known that wood smoke contains many compounds such as organic acids, phenols, and carbonyls which impart specific flavor to a food, improving organoleptic properties and simultaneously having antimicrobial activities against foodborne pathogens [50].

Fermented milk products are popular among consumers in Ethiopia and play important social, cultural, and economic roles [35]. Similar to other studies conducted in Ethiopia, this study found that fermentation was the most common means of processing milk into different dairy products. Fermenting milk produces organic acids which can reduce the pH of milk and further inhibit microbial growth, contributing to the microbiological safety of the products. Quantitative risk modeling by Makita et al. [51] showed that traditional fermentation of milk during the production of yoghurt can reduce the annual incidence rate of milk-borne staphylococcal poisoning in central Ethiopia by 93.7% (from 316 per 1000 without fermentation to 20 per 1000 with fermentation). However, acids produced through fermentation may not exert the same detrimental impacts on all pathogens in milk and milk products as some pathogens can survive the acidic environment and make the products unsafe for consumption [52]. Moreover, the milk products can be consumed before the fermentation process is completed and the pH sufficiently reduced, which can diminish the potential benefits of the process in controlling bacterial growth [53]. Therefore, under such situations, it should be noted that fermentation cannot be guaranteed to reduce milk-associated health risks unless a standardized method of fermentation is designed. Another risk mitigation practice identified in this study was the mixing of milk (especially goat milk) with boiling tea for consumption. This can be essentially considered a boiling process which can minimize potential milk-associated health hazards in the area.

Some respondents reported changes in their perception regarding the quality of milk and the associated health risks. These changes came about mainly through their participation in the existing health extension and livestock research activities in the area. For example, women who observed mastitis testing by researchers could explain the aspect of milk quality and the way their behavior changed. This suggests that practical demonstration of easily visible and credible diagnostic tests for milk quality assessment, such as alcohol and boiling tests in milk quality assessments, can be a useful strategy for effective awareness creation and may improve the understanding of the pastoralists regarding the biological mechanisms of food safety.

This study focused on women only, since they are the main actors in milk production and processing in the areas chosen for the study. However, other family members may also participate in dairy production and influence milk hygiene and safety. It might be useful for future studies in the same area to consider including these actors for a more comprehensive understanding of perceptions.

## Conclusion

This study tried to assess milk handling practices and perceptions and as such, integrated concepts from both the natural sciences (the practices of dairy production) and social sciences (the perception of people). Fischer et al. [40] recommend this kind of trans-disciplinary research approach to effectively mitigate food safety management in domestic environments. This study tried to uncover milk handling and processing practices and the consumption behaviors in a typical pastoral livestock keeping society. By employing participatory qualitative tools, it was possible to simultaneously identify milk handling practices which can negatively affect the health of the pastoralists and also risk mitigation strategies that potentially minimize milk-associated health illnesses. The findings highlight the need to promote hygienic practices through training and education specifically targeting the pastoralists and measuring the effect of these by closely engaging with local communities. Strengthening integration of milk hygiene in research and development programs can serve as an entry point for behavioral change towards safe handling and consumption of milk and milk products. Further studies are recommended to assess the interplay and cumulative effects of the risky behaviors and risk mitigation practices on health outcomes, potentially employing participatory risk modeling approaches.

## Abbreviations

FGD: Focus group discussion
IDI: In-depth interviews
